# When the Eagle Strikes Twice: A Case Report of Recurrent Stroke Secondary to Bilateral Stylocarotid Artery Syndrome

**DOI:** 10.7759/cureus.81266

**Published:** 2025-03-27

**Authors:** Walid Sadki, Naima Chtaou, Safae Farrai, Hamza Bouchacha, Youssef Alaoui Lamrani, Siham Bouchal, Aouatef El Midaoui, Abdellatif Oudidi, Mustapha Maaroufi, Mohammed Faouzi Belahsen

**Affiliations:** 1 Department of Neurology, Hassan II University Teaching Hospital, Fez, MAR; 2 Laboratory of Epidemiology and Research in Health Sciences, Sidi Mohamed Ben Abdellah University, Fez, MAR; 3 Department of Otolaryngology/Head and Neck Surgery, Hassan II University Teaching Hospital, Fez, MAR; 4 Department of Radiology, Hassan II University Teaching Hospital, Fez, MAR

**Keywords:** bony strokes, carotid dissection, eagle syndrome, elongated styloid process, stylocarotid artery syndrome

## Abstract

Eagle syndrome (ES), particularly its vascular variant known as stylocarotid artery syndrome (SAS), is characterized by an elongated styloid process (ESP) that impinges on the internal carotid artery (ICA), leading to vascular complications. We report a case of a 57-year-old man who presented with acute left-sided weakness. Imaging revealed right ICA dissection (ICAD), which was medically managed. Six years later, the patient presented with acute right-sided weakness, and imaging revealed a new left ICAD. Further review of the imaging findings identified bilateral ESP as the underlying cause of both dissections. The patient underwent a left-sided styloidectomy and had an uneventful recovery.

This case report describes a patient with vascular ES who experienced bilateral ICADs separated by several years. This underscores the importance of recognizing vascular ES as a potential and treatable cause of stroke.

## Introduction

Structural bone and cartilage anomalies affecting the arteries supplying the brain may serve as potential causes of ischemic stroke [1]. These rare entities are referred to as "bony strokes" [1]. In this context, ischemic strokes can arise through three pathophysiological mechanisms: (1) direct injury to the vessel wall, leading to endothelial damage or vessel dissection, which may result in artery-to-artery embolism; (2) chronic irritation of a vessel, promoting the formation of pseudoaneurysms, also known as dissecting aneurysms, which act as embolic sources; and (3) hemodynamic compromise of cerebral arteries due to vessel stenosis or occlusion [1]. Each of these mechanisms may be persistent or triggered by changes in the patient’s head position [1].

Among the various forms of bony stroke, Eagle syndrome (ES) represents a distinct subtype named after Watt W. Eagle, an otolaryngologist at Duke University who first described the classic variant in 1937 based on two cases [2]. It is characterized by an elongated styloid process (ESP) that may impinge upon or compress adjacent vasculo-nervous structures, most notably the internal carotid artery (ICA) [3]. ES has an incidence of four to eight per 10,000 individuals and may occur with or without abnormal angulation and/or ossification of the stylohyoid ligament [3]. Its vascular variant, known as stylocarotid artery syndrome (SAS), is even rarer [4] and occurs when an ESP directly affects the ICA, potentially leading to neurovascular conditions such as syncope, transient ischemic attacks (TIAs), or stroke [3]. A styloid process exceeding 2.5 cm is considered elongated, whereas a length of ≥3.0 cm is deemed clinically significant [5]. Although the incidence of an ESP in the general population is estimated to range between 4% and 7%, symptomatic cases account for only approximately 4% of individuals with this anatomical anomaly [6].

Although rare, ES is increasingly recognized as a cause of ischemic stroke, particularly in younger individuals without traditional vascular risk factors. This case report aims to raise awareness of vascular ES as an underdiagnosed yet significant cause of recurrent ischemic stroke.

## Case presentation

**Figure 1:**
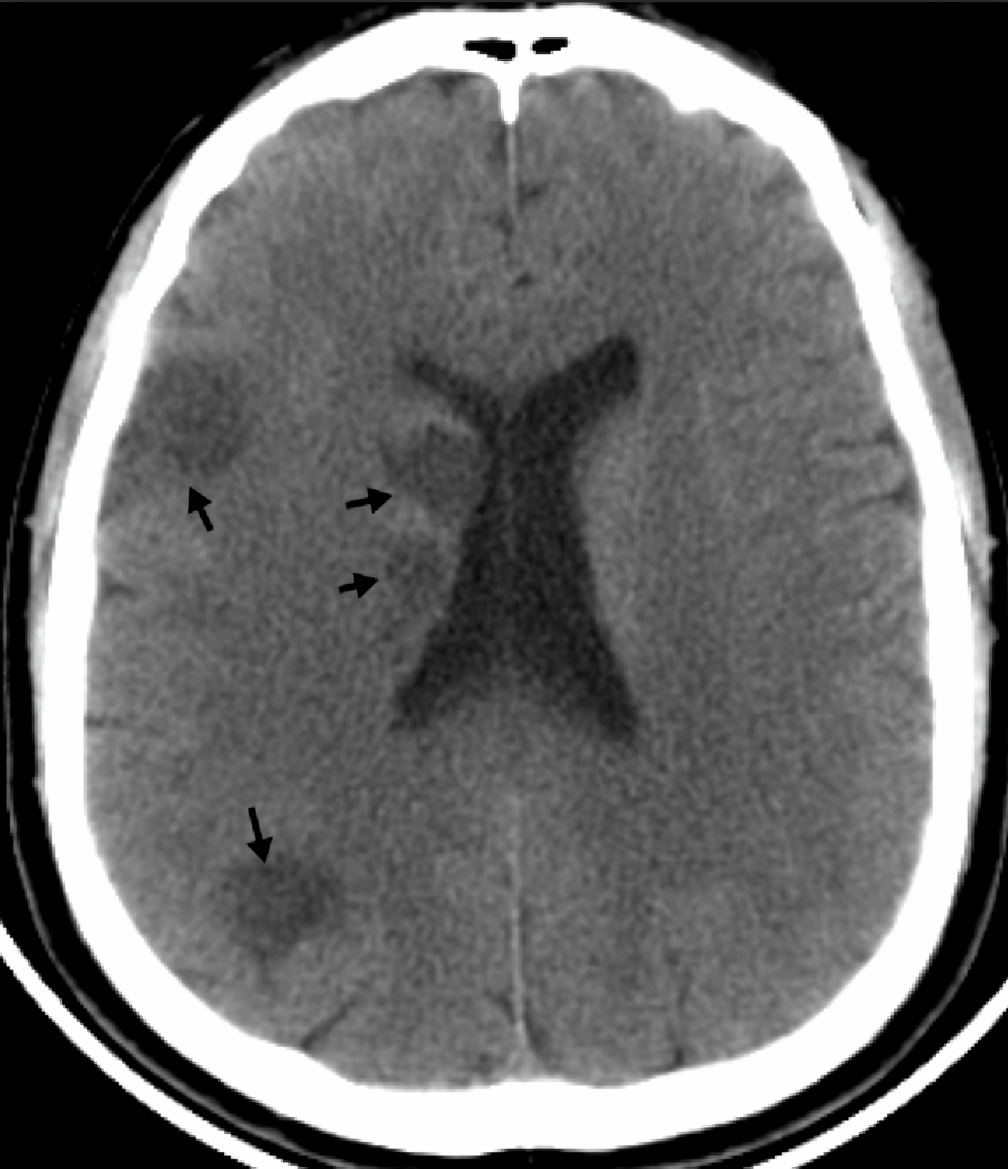
Non-enhanced computed tomography (CT) scan showed multiple subacute ischemic lesions (black arrows) within the right middle cerebral artery (MCA) territory.

A 57-year-old man presented to the emergency department in 2018 with sudden-onset left-sided limb weakness 30 hours after symptom onset. He had no traditional vascular risk factors and denied any previous trauma or significant medical history. Neurological examination at that time revealed mild dysarthria, left central facial palsy, left hemiparesis, and mild left-sided hypoesthesia, corresponding to a National Institutes of Health Stroke Scale (NIHSS) score of 8. Brain computed tomography (CT) showed multiple subacute ischemic lesions in the right middle cerebral artery (MCA) territory (Figure 1).

**Figure 2:**
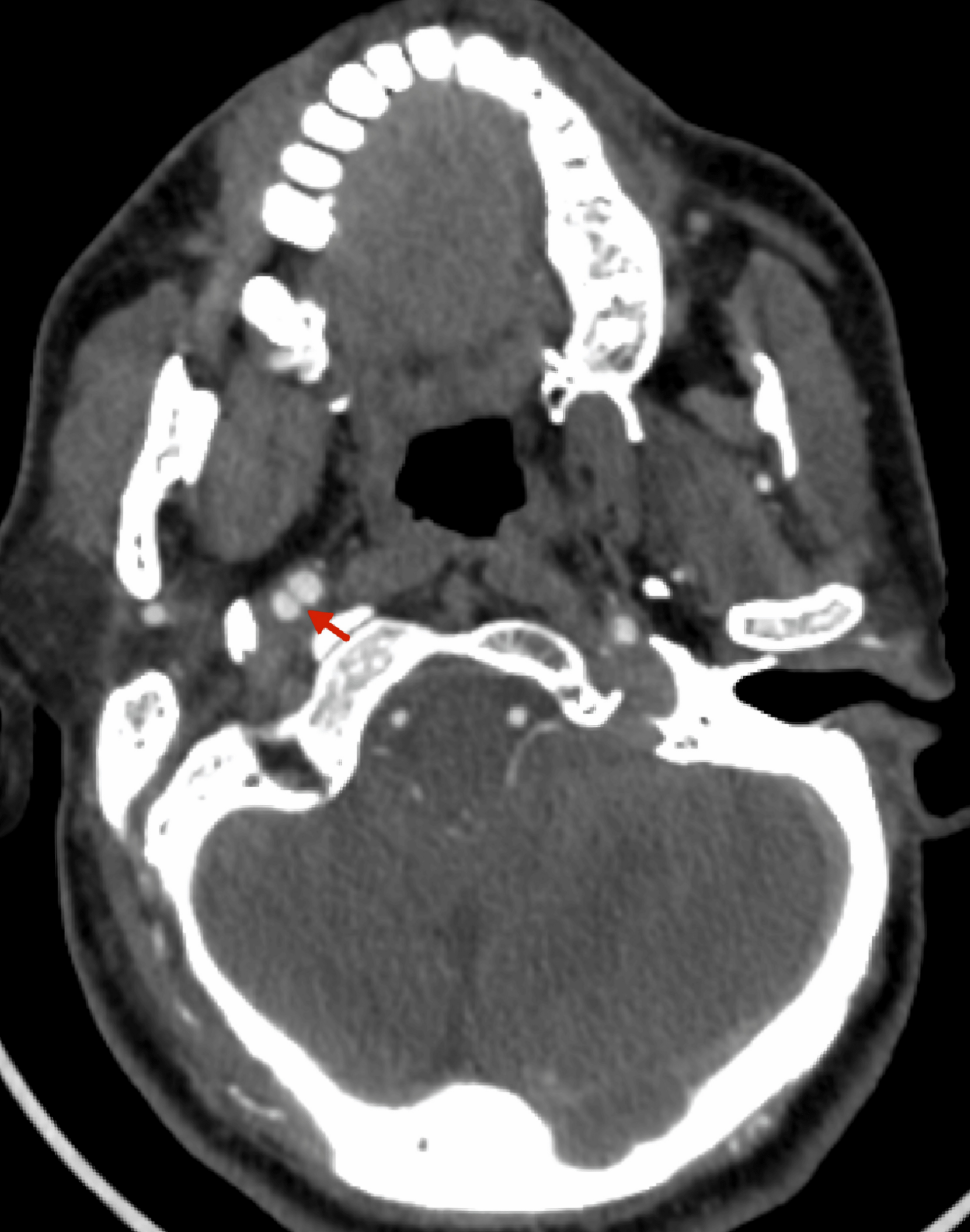
Computed tomography angiography (CTA) of the head and neck revealed an extracranial right internal carotid artery dissection, with the main feature being an intimal flap tear and a double lumen sign (red arrow), indicative of false lumen formation.

**Figure 3:**
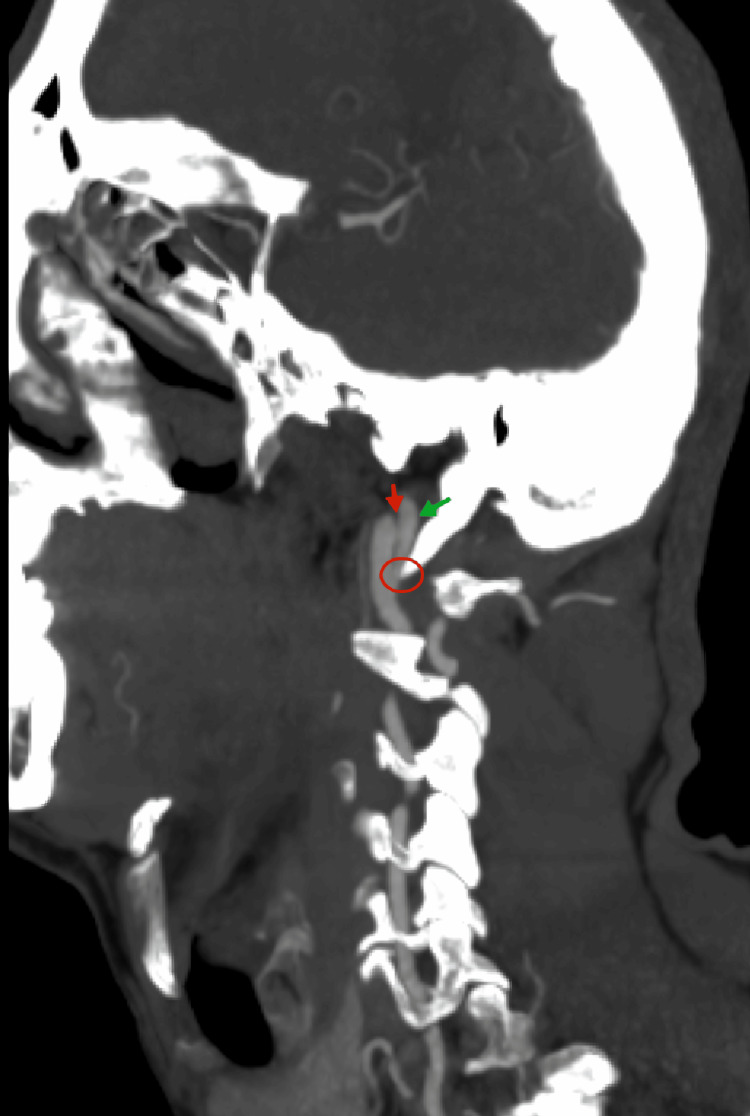
The computed tomography angiography (CTA) sagittal oblique view demonstrated an elongated styloid process (ESP) impinging on the right internal carotid artery (ICA) (indicated by the red circle), causing an intimal flap tear (red arrow) and a pseudoaneurysm (green arrow).

CT angiography (CTA) of the head and neck revealed extracranial right ICA dissection (ICAD), characterized by pseudoaneurysmal dilatation and an intimal flap tear (Figures 2, 3). Unfortunately, an ESP impinging on the ICA (Figure 3) was not identified at that time. The patient was discharged after rehabilitation with a single-antiplatelet regimen of aspirin (100 mg per day) due to the absence of high-risk radiographic features predicting ischemic stroke after dissection, such as occlusion or intraluminal thrombus. His recovery was favorable, with mild spasticity and a modified Rankin Scale (mRS) score of 1. One month later, he developed post-stroke epilepsy, which was well controlled with carbamazepine. Over the following six years, the patient remained clinically stable without recurrent ischemic events.

**Figure 4:**
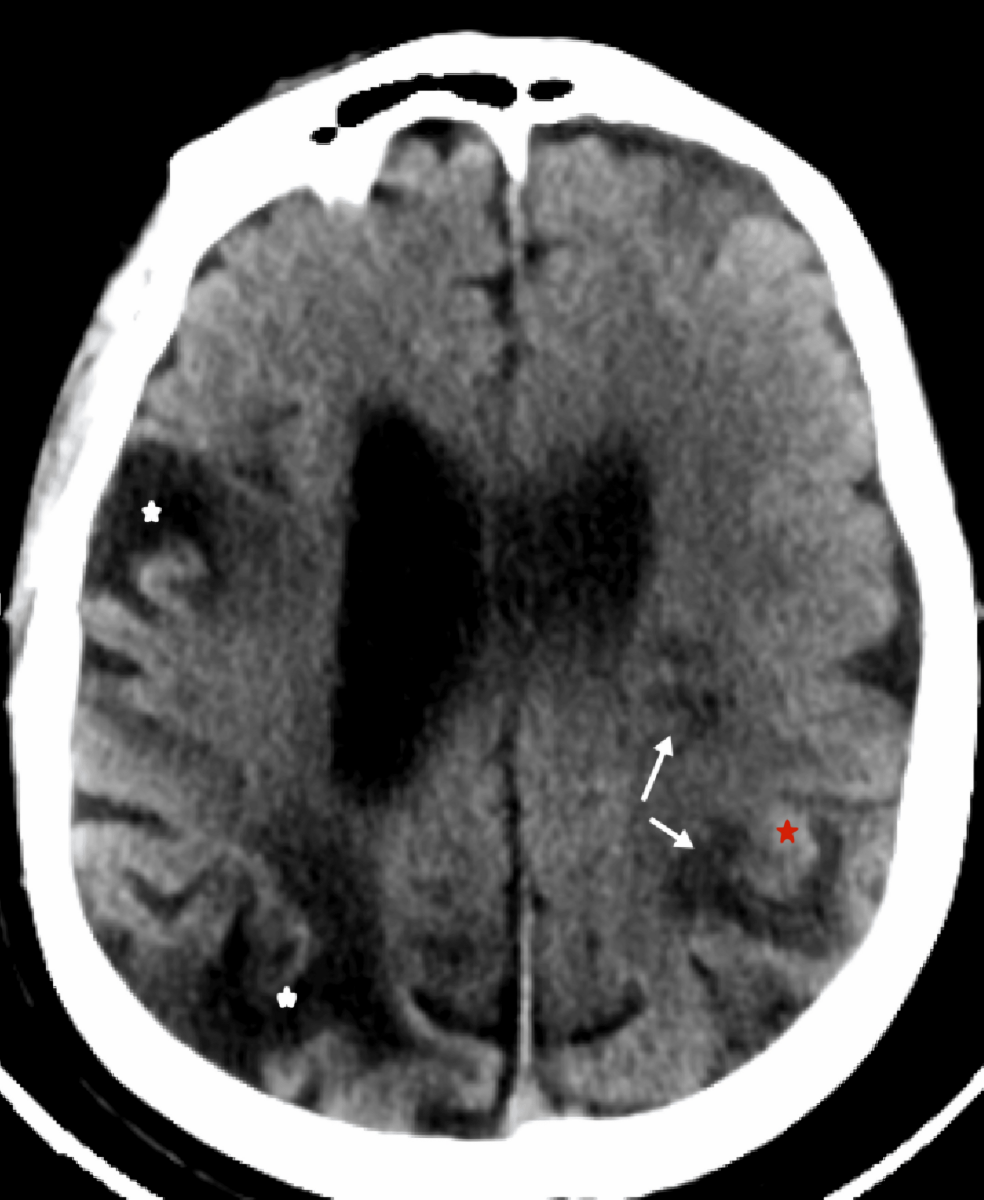
Non-contrast computed tomography (CT) scan showed subacute ischemic lesions in the left middle cerebral artery (MCA) territory (white arrows), with hemorrhagic transformation (red star) and sequelae of right MCA infarction (white stars).

Six years later, the patient was readmitted to the emergency department with sudden-onset right-sided limb weakness 28 hours after symptom onset. Neurological examination revealed severe dysarthria, right central facial palsy, right hemiplegia, transient left-sided Horner's syndrome, and mild left-sided spasticity persisting from the first stroke, corresponding to an NIHSS score of 12. A brain CT scan showed subacute ischemic lesions in the left MCA territory (Figure 4).

**Figure 5:**
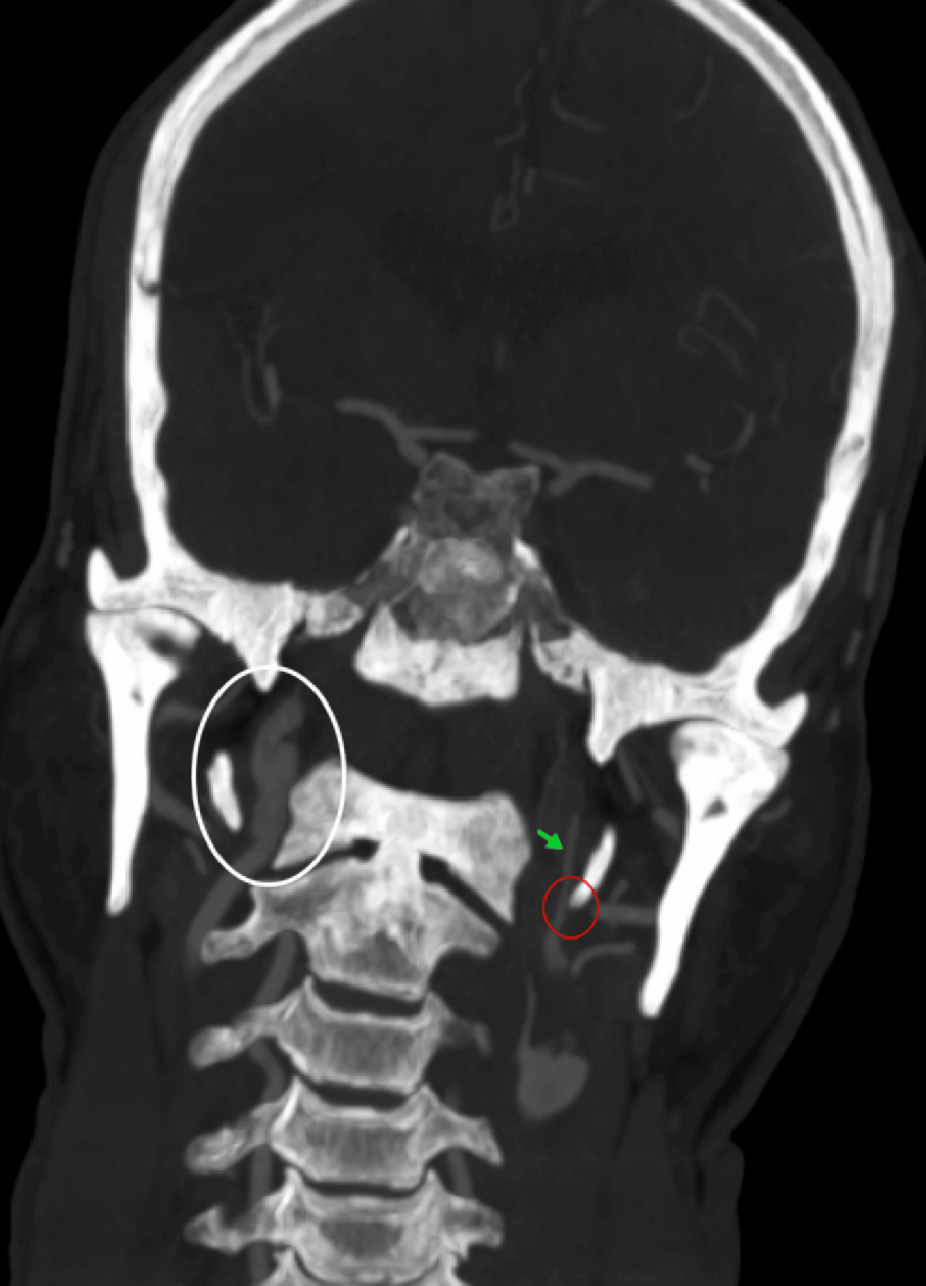
Computed tomography angiography (CTA) of the head and neck revealed a new left internal carotid artery (ICA) dissection (green arrow), resulting in severe stenosis of the ICA caused by the impinging elongated styloid process (ESP) (red circle). Persistent involvement of the contralateral ICA, including dissection and conflict with the right ESP, is shown within the white circle.

**Figure 6:**
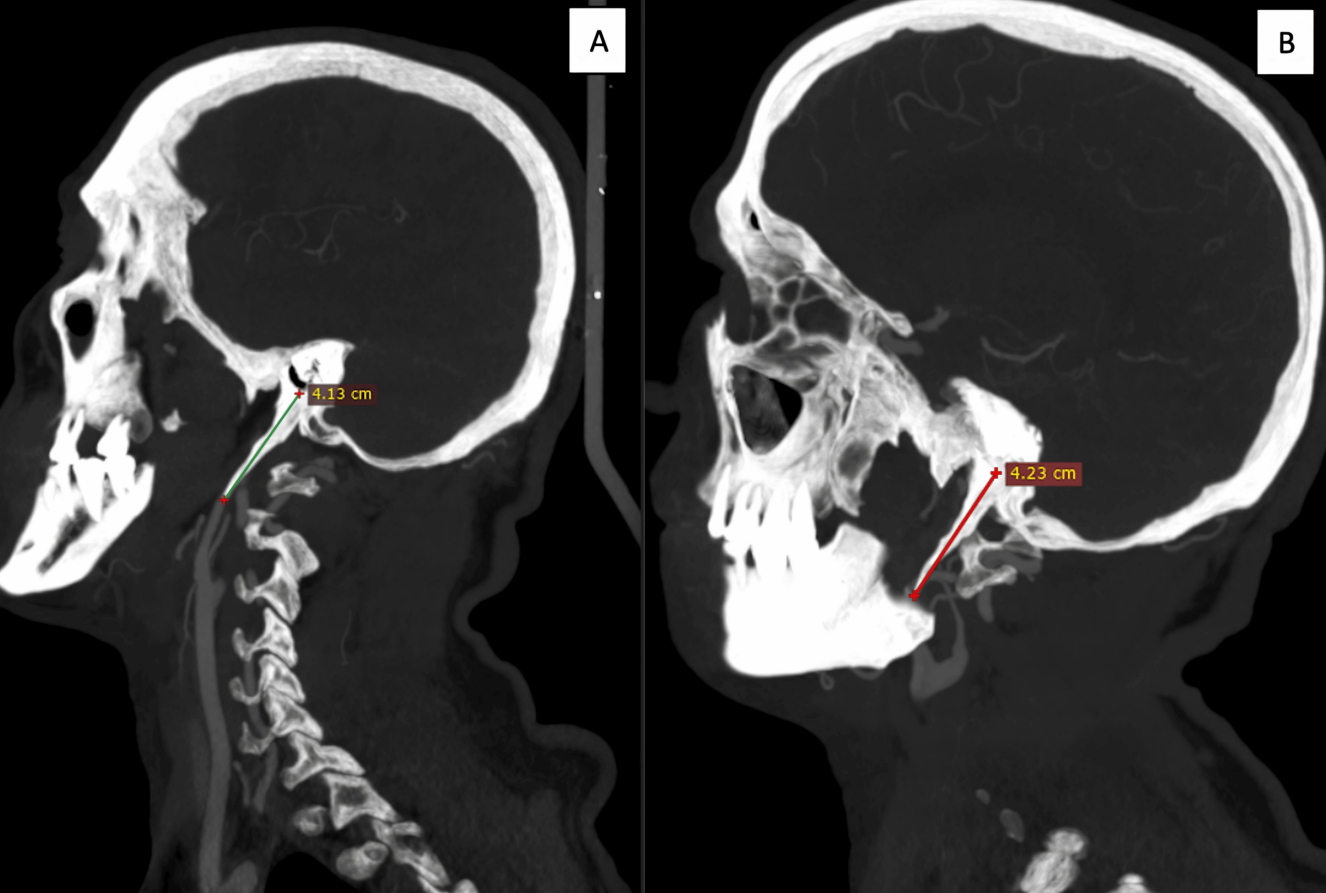
Bilateral elongated styloid processes (ESPs), measuring 4.13 cm on the right (A) and 4.23 cm on the left (B).

CTA of the head and neck revealed a new left ICAD causing severe stenosis of the cervical segment (Figure 5). Further review of the images identified a left ESP directly impinging on the left ICA, along with persistent involvement of the contralateral ICA (Figure 5). Both vascular abnormalities were attributed to bilateral ESPs (each measuring >4 cm), resulting in a direct insult to the walls of the ICAs (Figure 6).

**Figure 7:**
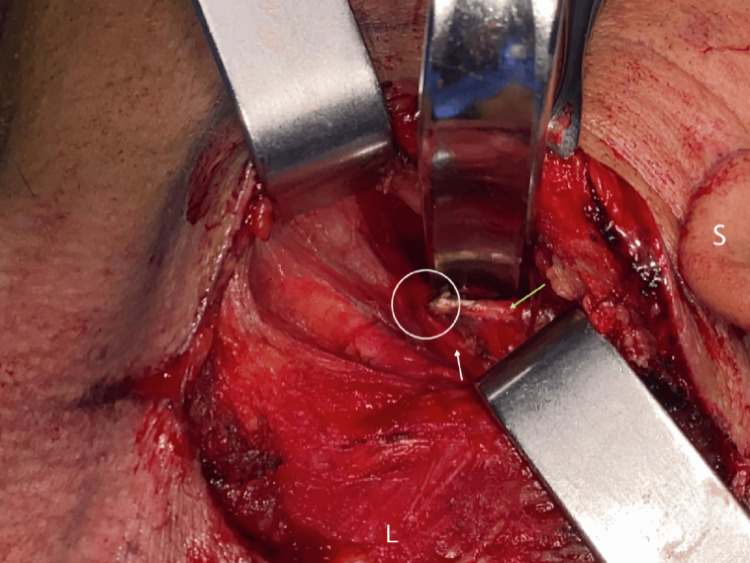
Intraoperative photograph confirming direct injury (white circle) to the vessel wall of the left internal carotid artery (ICA) (white arrow), caused by the elongated styloid process (ESP) (green arrow). L: left; S: superior

**Figure 8:**
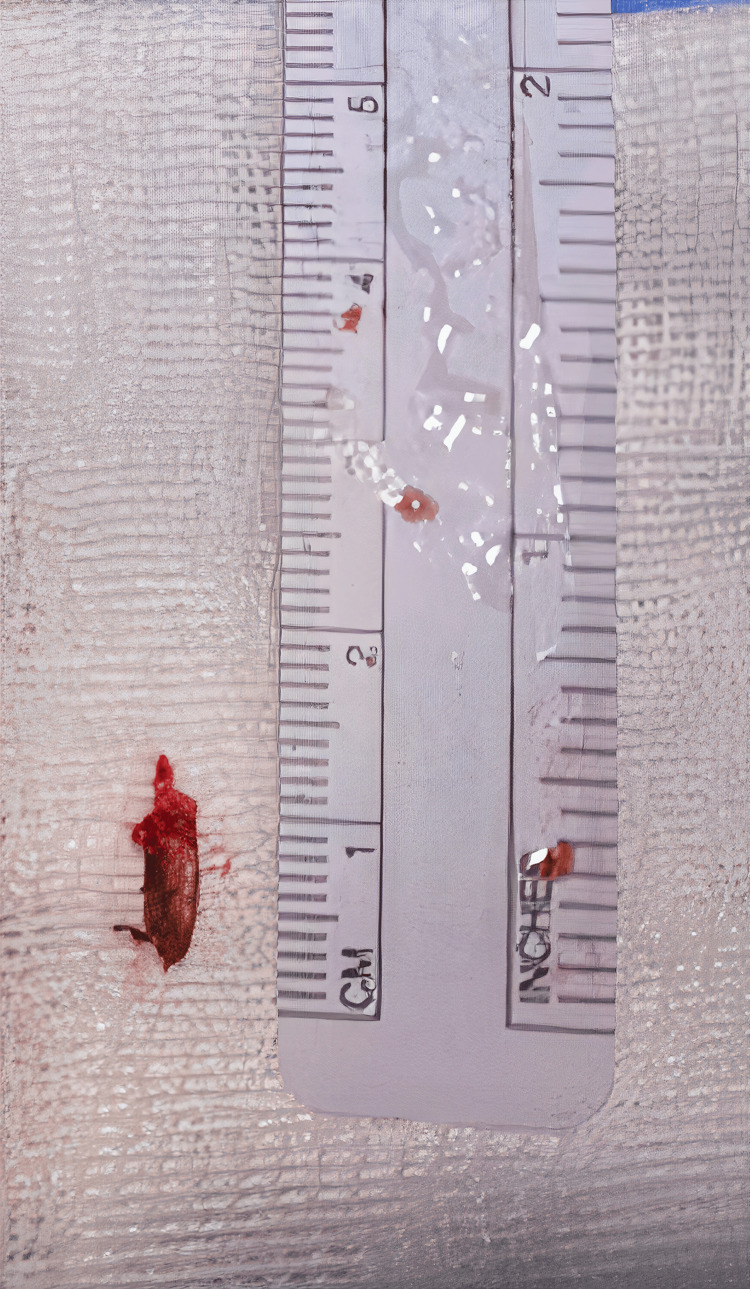
Transected left styloid process following transcervical styloidectomy (approximately 1 cm resection).

Seven days post-stroke, the patient underwent transcervical styloidectomy for left-sided ESP. Intraoperative findings confirmed a direct insult to the vessel wall of the left ICA caused by ESP (Figure 7). The transected left styloid process following transcervical styloidectomy is shown in Figure 8.

The post-operative course was uneventful, and the patient was discharged on a single antiplatelet regimen, based on the absence of intraluminal thrombus or occlusion in the left ICA and the identification of hemorrhagic transformation within the ischemic cerebral lesion. Three months later, only moderate improvement was observed, although Horner's syndrome showed improvement following styloidectomy. The mRS score at that time was 4, indicating that the patient required assistance for ambulation. No further ischemic or neurological events occurred during this period. Resection of the right ESP will be scheduled within six months after the left styloidectomy, as the patient remained asymptomatic on the right side, with no further clinical events over the following six years and no significant changes in the radiological appearance of the right ICAD, particularly with the pseudoaneurysm diameter remaining stable.

## Discussion

The styloid process is a slender elongated projection of the temporal bone. It originates at the junction of the petrous base and mastoid process, extending anteroinferiorly. It is positioned between the internal and external carotid arteries and is located near the internal jugular vein and cranial nerves V, IX, X, and XII.

Consequently, ES may lead to a variety of symptoms, including throat pain, dysphagia, and foreign body sensations [1,2]. The syndrome is typically classified into two main variants: the classic variant, which involves compression of adjacent nerves, and the vascular variant termed SAS, which involves compression of adjacent vessels, particularly the ICA [7]. This case demonstrates bilateral ICAD secondary to the vascular variant of ES, a rare condition that leads to recurrent stroke, as reflected in the title “When the Eagle Strikes Twice.”

SAS was later characterized by Watt W. Eagle in 1949 [8]. This vascular variant of ES is of particular clinical concern due to its potential to compromise the ICA. It can cause vascular compression, injury to vessel walls leading to dissection and embolization, or vasovagal response [9]. Vascular compression is frequently intermittent and commonly precipitated by neck rotation. This can occur either directly because of the close proximity of the ESP to the ICA or indirectly via the stylopharyngeus muscle [9]. Although a vasovagal response component cannot be ruled out, this mechanism can lead to TIAs or syncope during neck rotation.

In the present case, the mechanism of stroke was direct vessel injury leading to cervical ICAD. This vascular insult can cause intimal tearing, resulting in the formation of a pseudoaneurysm, intramural hematoma, and subsequent luminal narrowing or occlusion [10]. A direct correlation has been noted between the increasing length of the styloid process, its proximity to the ipsilateral ICA, and the likelihood of developing cervical ICAD [11]. Specifically, the risk of cervical ICAD increases by an odds ratio of 1.08 for every 1 mm increase in the length of the styloid process [11]. This mechanism can also be triggered or exacerbated by musculoskeletal disorders, trauma, neck massage, or extreme head and neck movement. These factors highlight the importance of performing dynamic CTA or magnetic resonance angiography (MRA) with varying head positions to ensure accurate diagnosis [12]. However, in many cases, including ours, the absence of these triggers suggests that they are not always substantial contributors.

Two recent reviews of ischemic stroke associated with SAS identified 23 cases reported between 2014 and 2024 [13] and 29 patients based on a 2023 database search [14]. Notably, both studies demonstrated a strong male predominance, with a mean age of approximately 48-51 years, although a wide age range was observed [13,14]. Patients with SAS present with nonspecific symptoms, including hemiplegia, aphasia, neck pain, and Horner's syndrome. In our patient, the symptoms were similarly nonspecific, aside from Horner's syndrome, which may suggest underlying ICAD. Imaging findings typically include focal cortical infarcts due to embolization of distal branches of the MCA [14]. In most cases, both ESP and ischemic strokes are bilateral [13,14], with ICAD identified as the primary underlying mechanism [13,14]. Notably, bilateral ICAD was observed in 26% of patients [13], a finding consistent with our patient’s presentation, thereby underscoring the importance of bilateral evaluation. In terms of diagnostic tools, imaging, particularly reconstructed three-dimensional CTA and MRA, plays a crucial role in SAS diagnosis by delineating the accurate spatial relationship between the ESP and ICA as well as identifying vascular complications such as dissection or stenosis [15]. In this case, CTA revealed bilateral ICAD, demonstrating irregular luminal narrowing in the left ICA, intimal flap, and pseudoaneurysm (focal arterial wall dilatation) in the right ICA (Figures 2, 3, 5).

The management of patients with SAS requires an interdisciplinary approach. Owing to the rarity of the condition, established guidelines for treatment have yet to be defined. From a pathophysiological perspective, styloidectomy remains the definitive treatment [16]. This procedure can be performed using two approaches: intraoral (transoral) or extraoral (transcervical). The intraoral approach avoids external scarring but is associated with limited surgical field visibility and the risk of deep neck space infections. In contrast, the transcervical approach, which is increasingly favored, offers improved visibility, a lower risk of deep neck infections, and the ability to remove a larger portion of the styloid process [16]. Moreover, the transcervical technique, as performed in this case, has become so refined that the incision is minimal, and post-operative recovery is rapid. Although endovascular management is well tolerated for ICAD in general, stenting alone is not a definitive treatment for ES. It should be combined with styloidectomy [16-18]. The surgical approach addresses the underlying neurovascular conflict and mitigates risks, such as stent fracture or distal migration caused by persistent mechanical stress, which can lead to vascular complications or recurrent stroke [17-19]. Furthermore, contralateral styloidectomy can mitigate the risk of further neurological injury in cases of bilateral ESP [16].

In our case, the right styloidectomy is planned within six months following the left procedure. This staged approach was chosen due to the patient’s residual neurological deficits after the recent stroke, which required time for recovery. Such an approach, commonly used in similar cases [16], reduces surgical risks and allows for more controlled recovery. By adopting this strategy, we aim to minimize complications and ensure optimal neurological recovery before proceeding with the right styloidectomy.

## Conclusions

In conclusion, the present case underscores the importance of considering ES, albeit rarely, in the diagnostic evaluation of cerebrovascular events. Comprehensive imaging is essential for assessing both the vascular lumen and adjacent anatomical structures. Early diagnosis and timely intervention are critical for preventing severe complications such as stroke. Advances in imaging techniques and the availability of surgical options have significantly improved the prognosis of patients with this uncommon condition.
